# A domain adaptation benchmark for T1-weighted brain magnetic resonance image segmentation

**DOI:** 10.3389/fninf.2022.919779

**Published:** 2022-09-23

**Authors:** Parisa Saat, Nikita Nogovitsyn, Muhammad Yusuf Hassan, Muhammad Athar Ganaie, Roberto Souza, Hadi Hemmati

**Affiliations:** ^1^Electrical and Software Engineering, Schulich School of Engineering, University of Calgary, Calgary, AB, Canada; ^2^Centre for Depression and Suicide Studies, St. Michael's Hospital, Toronto, ON, Canada; ^3^Mood Disorders Program, Department of Psychiatry and Behavioural Neurosciences, McMaster University, Hamilton, ON, Canada; ^4^Electrical Engineering, Indian Institute of Technology, Gandhinagar, Gujarat, India; ^5^Chemical Engineering, Indian Institute of Technology, Kharagpur, West Bengal, India; ^6^Hotchkiss Brain Institute, Cumming School of Medicine, University of Calgary, Calgary, AB, Canada; ^7^Electrical Engineering and Computer Science, Lassonde School of Engineering, York University, Toronto, ON, Canada

**Keywords:** deep learning, domain adaptation, magnetic resonance imaging, neuroimaging, segmentation, brain

## Abstract

Accurate brain segmentation is critical for magnetic resonance imaging (MRI) analysis pipelines. Machine-learning-based brain MR image segmentation methods are among the state-of-the-art techniques for this task. Nevertheless, the segmentations produced by machine learning models often degrade in the presence of expected domain shifts between the test and train sets data distributions. These domain shifts are expected due to several factors, such as scanner hardware and software differences, technology updates, and differences in MRI acquisition parameters. Domain adaptation (DA) methods can make machine learning models more resilient to these domain shifts. This paper proposes a benchmark for investigating DA techniques for brain MR image segmentation using data collected across sites with scanners from different vendors (Philips, Siemens, and General Electric). Our work provides labeled data, publicly available source code for a set of baseline and DA models, and a benchmark for assessing different brain MR image segmentation techniques. We applied the proposed benchmark to evaluate two segmentation tasks: skull-stripping; and white-matter, gray-matter, and cerebrospinal fluid segmentation, but the benchmark can be extended to other brain structures. Our main findings during the development of this benchmark are that there is not a single DA technique that consistently outperforms others, and hyperparameter tuning and computational times for these methods still pose a challenge before broader adoption of these methods in the clinical practice.

## 1. Introduction

Magnetic Resonance Imaging (MRI) has demonstrated its exceptional utility for clinical research in neurology and psychiatry (Nishimura, 2010). Brain segmentation is a fundamental method that provides a wide array of information about the brain structure in neuroimaging. With image segmentation, researchers can measure the volume of a particular brain region to characterize, for example, patterns of healthy brain aging (Valizadeh et al., 2017), the impact of neurodegenerative diseases (Sander et al., 2019), and yield clinically meaningful information that may potentially guide treatment selection for patients with depression (Nogovitsyn et al., 2020).

**Figure 1:**
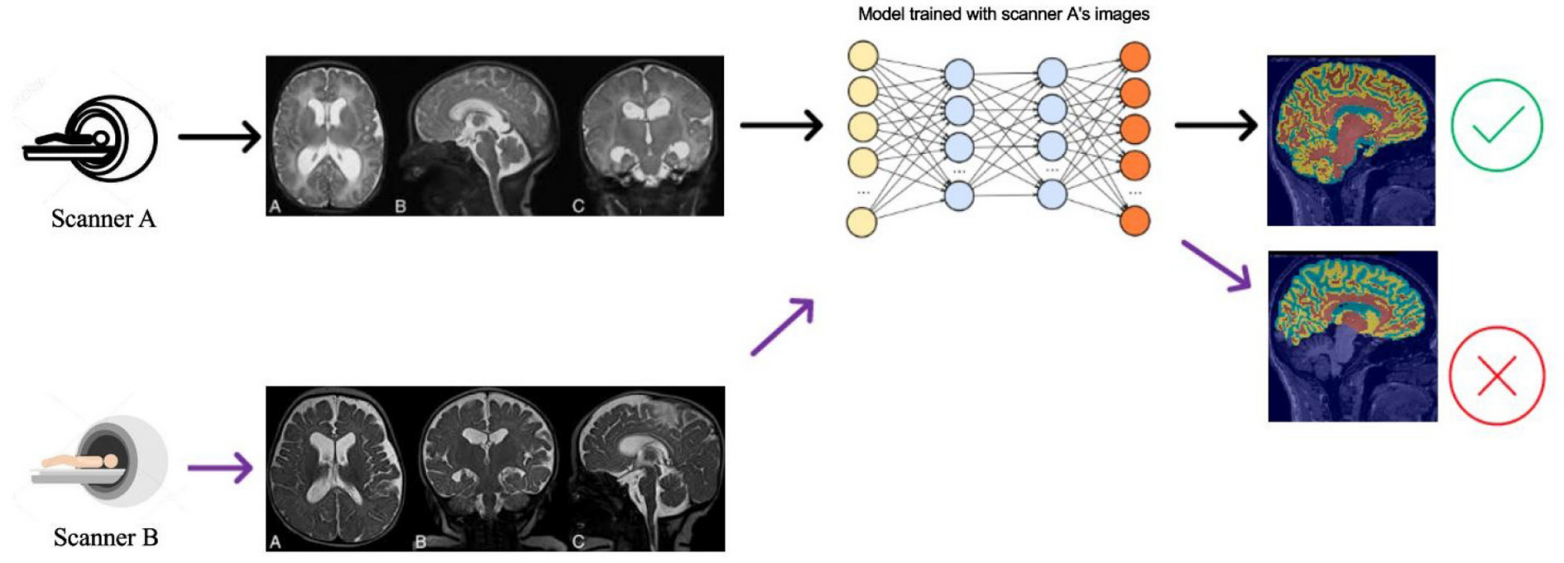
Illustration of the DA problem. Deep learning segmentation models often do not work well when there is a data distribution shift between the source (Scanner A) and the target (Scanner B) data distributions.

Deep learning algorithms are the current state-of-the-art for brain MRI segmentation (Pereira et al., 2016; Thyreau et al., 2018; Kuijf et al., 2019; Henschel et al., 2020). These algorithms hold promise for future applied neuroimaging because of their unparalleled processing speed and accuracy (Henschel et al., 2020). Nevertheless, deep learning models can suffer from generalization problems (Bento et al., 2021). For example, a brain MRI segmentation model trained with a particular source dataset can provide accurate segmentation results for test samples from the same dataset. Still, if used on a new target dataset, the segmentation algorithm may show compromised performance with inaccuracies in volumetric measures (Figure 1). This happens because of data distribution differences between the source and test data—and is known as the domain shift problem.

Domain shifts in the data are expected to occur over time. For example, in the early 2000s, most MRI scanners had 1.5 T magnetic field strength. Nowadays, in most developed countries, the standard has moved to 3 T. MR images acquired using 3 T scanners have a higher signal-to-noise ratio (SNR) than images from 1.5 T scanners. Still, it is uncertain whether models trained using data collected 1.5 T scanners will generalize to 3 T scanners. Other common sources of data variability that result in domain shifts are the use of different receiver coils, image acquisition protocols, and reconstruction algorithms like Parallel Imaging (Deshmane et al., 2012), Compressed Sensing (Lustig et al., 2008), and other machine learning methods (Beauferris et al., 2022). Also, MR images produced by different scanners differ depending on the scanner vendor because differences in hardware and software impact their data distribution.

Domain shift is a significant problem that can degrade the performance of deep-learning-based segmentation models. Domain adaptation (DA) techniques (Perone et al., 2019) can be used to adapt models that were trained using a source dataset to a target dataset. The capacity to adjust brain MRI segmentation models to new data in the presence of domain shifts is essential, especially when scientists attempt to translate these models from research to clinical practice.

DA methods can be classified as supervised and unsupervised methods. Supervised DA requires a set of labeled data in the target domain. The most common approach for supervised DA is fine-tuning a pre-trained model. Specially in medical image analysis, where often limited labeled data is available, fine-tuning Convolutional Neural Networks (CNNs) can show a performance as good as or even better than CNNs trained from scratch. However, fine-tuning results depends on the application, the amount of data available, and the models being used (Tajbakhsh et al., 2016). Several studies have examined fine-tuning approaches for medical image analysis (Van Opbroek et al., 2014; Cheplygina et al., 2017; Ghafoorian et al., 2017; Xu et al., 2017; Dou et al., 2018; Ataloglou et al., 2019; Valverde et al., 2019; Shirokikh et al., 2020).

Unsupervised DA corresponds to DA methods that do not use labeled target data. Unsupervised DA methods attract more attention in image segmentation research as it does not require time consuming manual data labeling (Kouw and Loog, 2019). Several unsupervised domain adaptation techniques have been proposed over the years (Menze et al., 2014; Kamnitsas et al., 2017; Shrivastava et al., 2017; Gholami et al., 2018; Li et al., 2019; Perone et al., 2019; Yan et al., 2019; Ackaouy et al., 2020).

Generative Adversarial models have recently been proposed for DA purposes. These adversarial models either utilize image translation from one domain to another as a preprocessing step before employing a pretrained segmentation model, or they use a one-step end-to-end model that combines image translation and segmentation. Some of these methods need paired data, i.e., the same image in both domains (Armanious et al., 2020). These strategies can work in a supervised and unsupervised setting. A few strategies work without paired data and have supervised and unsupervised versions (Zhu et al., 2017; Oliveira et al., 2020).

A current issue is that with the current lack of benchmarks for objective comparisons between methods, there is no established way of choosing the best among these techniques (cf., Guan and Liu, 2021). Another concern in evaluating and comparing existing domain adaptation approaches for medical image segmentation is that many of these approaches have only been validated on private datasets or on datasets that were not specifically designed for domain adaptation purposes. Addressing this issue is one of the objectives of the CrossMoDA (Cross-Modality Domain Adaptation) challenge (Dorent et al., 2022). The 2021 CrossMoDA challenge was held with the aim of presenting a benchmark of unsupervised domain adaptation methods for vestibular schwannoma and cochlea segmentation, which is a problem with limited practical applicability. Also, the goal of the CrossMoDA challenge was to investigate whether fewer than normally acquired MRI sequences could yield equivalent information about the tumor being investigated, resulting in shorter and cheaper MRI exams. Another interesting DA benchmark was proposed in Campello et al. (2021), but applied to cardiac images and not brain segmentation.

In this work, we propose a benchmark that compares different DA techniques for T1-weighted brain MR image segmentation. Our benchmark uses the Calgary-Campinas (CC) dataset (Souza et al., 2018) that has images collected from three MRI scanner vendors (Philips, Siemens, General Electric [GE]). Our benchmark currently focuses on two segmentation tasks: (1) skull-stripping which is to isolate brain tissue from non-brain tissue, and (2) white-matter (WM), gray-matter (GM), and cerebrospinal fluid (CSF) segmentation. These tasks are often the initial processing steps in many neuroimaging pipelines. Moreover, the benchmark framework that we propose here can be extended to other brain structures. The main contributions of our work are as follows:

- Proposal of a benchmark for objectively evaluating different DA techniques for brain MR image segmentation.
- A public repository with code scripts for developing baseline segmentation models and extraction of quantitative metrics^1^.
- Addition of data annotation to the CC dataset.
- Creation of a public leaderboard comparing different DA approaches ^2^.

## 2. Data

### 2.1. T1-weighted MRI data

**Figure 2:**
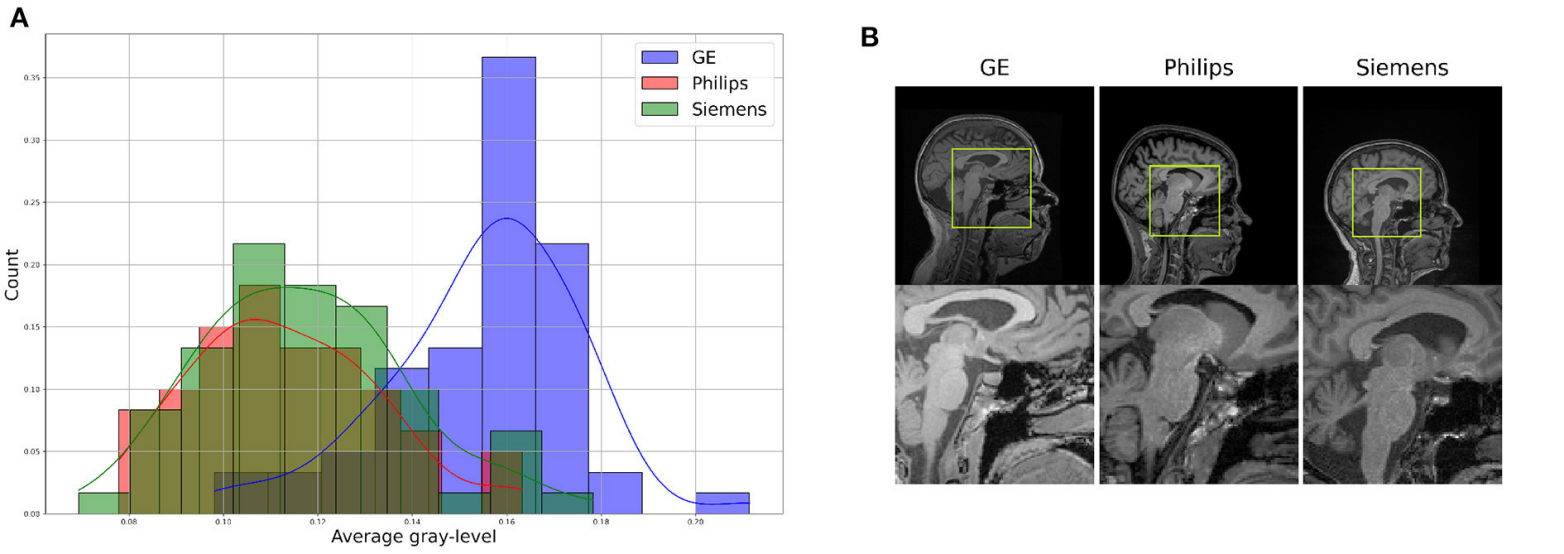
Illustration of differences between the domains, which can be see through **(A)** a difference in data distribution (after scaling data between 0 and 1), and **(B)** a visual comparison of similar brain regions across domains.

For the proposed benchmark, we use the Calgary-Campinas-359 (CC-359) dataset (Souza et al., 2018). The CC-359 is a multi-vendor, multi-field strength volumetric brain MRI dataset. The CC-359 has data acquired in scanners from three different vendors (GE, Philips, and Siemens) at both 1.5 and 3 T magnetic field strengths. We only use 3 T data since 1.5 T scanners have been replaced with 3T scanners with higher imaging quality in most facilities, and we want to have a manageable number of domains for the benchmark. The 3 T portion of the dataset comprises 180 T1-weighted three-dimensional (3D) volumes [3D MP-RAGE (Philips, Siemens), and a comparable T1-weighted spoiled gradient echo sequence (GE)], with 60 subjects per vendor. The volumes are collected from 180 (50.00% female) presumed healthy subjects with an age range from 29 to 80 years, and 53.4 ± 7.9 years (*mean*±*std*). In this benchmark, we consider each scanner vendor as a different domain (i.e., three domains). We chose these three domains because of varying image distributions across vendor-specific hardware and software differences, which inherently lead to domain shifts in the data (Figure 2). To support this claim, we show in the Supplementary material that a simple convolutional neural network model achieves 99.44 ± 0.79% (*mean*±*std*) accuracy when distinguishing between images acquired using MRI scanners of different vendors. A summary of the dataset is shown in Table 1.

**Table 1:** Dataset summary.

**Scanner**	**Age**	**Gender**	**Train**	**Validation**	**Fine-tuning**	**Test**
GE	53.6 ± 5.7	30 M/30 F	38	10	12	12
Philips	50.3 ± 9.3	30 M/30 F	38	10	12	12
Siemens	56.6 ± 6.9	30 M/30 F	38	10	12	12

Columns from left to right: scanner, average age (mean ± standard deviation), gender (number of male/number of female subjects), train, validation, fine-tuning, and test sets (number of volumes). The 12 subjects used for fine-tuning come from the target domain.

### 2.2. Brain masks

The CC-359 dataset provided brain masks for all its brain volumes. The masks were obtained by using the Simultaneous Truth and Performance Level Estimation (STAPLE) algorithm (Warfield et al., 2004) to combine the brain masks obtained with eight publicly available skull-stripping techniques: Advanced Normalization Tools (ANTs) (Avants et al., 2011), Brain Extraction based on non-local Segmentation Technique (BEaST) (Eskildsen et al., 2012), Brain Extraction Tool (BET) (Smith, 2002) from FSL (Jenkinson et al., 2012) software, Brain Surface Extractor (BSE) (Shattuck et al., 2001) from BrainSuite (Shattuck and Leahya, 2002) software, Hybrid Watershed Approach (HWA) (Ségonne et al., 2004) from Freesurfer (Dale et al., 1999) software, Marker Based Watershed Scalper (MBWSS) (Beare et al., 2013), Optimized Brain Extraction (OPTIBET) (Lutkenhoff et al., 2014), and Robust Brain Extraction (ROBEX) (Iglesias et al., 2011). The STAPLE masks were reviewed by a medical expert to assure the quality of the segmentation.

### 2.3. WM, GM, and CSF masks

As part of this benchmark, we included WM, GM, and CSF masks for all brain volumes of the CC-359 dataset. The masks were obtained by using the STAPLE algorithm to combine the segmentation obtained with the Automated Segmentation Tool (Zhang et al., 2000) from FSL and the Unified Segmentation method (Ashburner and Friston, 2005) from the software Statistical Parameter Mapping (Flandin and Friston, 2008). A medical expert reviewed the STAPLE masks to assure the quality of the segmentation.

## 3. Preprocessing and data augmentation

For consistency, the same preprocessing and data augmentation strategy for all methods in our benchmark. In this subsection, we present this strategy.

To account for the different ranges of pixel intensities across datasets, we preprocess the images using min-max normalization to assure all images have the same intensity range. The normalization is done per MRI volume following the equation:


(1) $$\begin{array}{l} V_{norm}=\frac{V-min\left(V\right)}{max\left(V\right)-min\left(V\right)}, \end{array}$$

where V represents the 3D volume we are normalizing.

Working with 3D images requires more memory to load the data and more computational power to train models. Lucena et al. (2019) reported that the segmentation performance had marginal improvement when using a tri-planar approach as opposed to using a single image plane to train the segmentation model. Due to this observation and the fact that 3D models can be too computationally intensive for training, we opted for employing 2D models trained using 2D slices. We used the sagittal plane for slice extraction since it is the plane where the best results were obtained by Lucena et al. (2019).

Empty slices (i.e., with no signal) were removed to reduce the computational burden during model training. We also applied data augmentation transformations including random rotation, shift, scaling, and cropping on 2D slices using a fast open source library named Albumentation (Buslaev et al., 2020). We used patches of size 128 × 128 to train the baseline and fine-tuning models and used the whole images of size 288 × 288 to train the self-ensembling and unlearning models. Table 2 shows the number of training slices in each data domain after preprocessing was applied.

**Table 2:** Summary of number of slices per domain, per train, validation, and fine-tuning sets after preprocessing of the volumes.

**Scanner**	**Train**	**Validation**	**Fine-tuning**
GE	9,392	2,016	2,400
Philips	8,640	1,786	2,160
Siemens	10,752	2,237	2,688

The test metrics are computed per volume so the number of test slices is not relevant and omitted here.

## 4. Methods

This section describes the experimental setup and quantitative metrics to evaluate the different DA models. We also describe the models included in our benchmark.

### 4.1. Data split

The proportion of data in each train and test set is based on the common percentages used in machine learning problems which are 80% for the train set and 20% for the test set. The train set itself was split randomly to 80% for the train and 20% for validation. The proposed data split resulted in 12 volumes in the test for each domain in the benchmark, which should be sufficient to detect changes in segmentation performance across domains. The training, validation, and test files are provided in the benchmark repository to make experiments reproducible. We also reserve 20% of volumes in each domain train set for the supervised DA methods that require labeled data. A summary of the data split is shown in Table 1.

### 4.2. Evaluation metrics

To evaluate the performance of different segmentation models in the benchmark, we compare the segmentation masks predicted by each model with the ground truth masks for skull-stripping and WM, GM, and CSF segmentation. We calculated two metrics commonly used for benchmarking segmentation methods (Timmins et al., 2021): the Dice similarity coefficient (Yeghiazaryan and Voiculescu, 2015) and the 95th percentile Hausdorff distance (HD) (Taha and Hanbury, 2015). These metrics were selected from two common categories for medical image segmentation evaluation: overlap metrics (Dice coefficient) which measures the total overlap of ensembles of labels defined on multiple test images, and a complementary measure of error, the boundary distance, which is an indicator of non-overlapping segmentation contours (Crum et al., 2006). Suppose that *G* is the ground truth image and *S* is the segmentation we want to assess, the Dice coefficient and Hausdorff distance metrics are given by the following equations:

- Dice coefficient:

(2) $$\begin{array}{l} Dice=\frac{2*|S\cap G|}{\left|S|+|G\right|} \end{array}$$

- Haussdorff distance:

$$d_{H}(S,G)=\max\{\underset{s\in S}{\sup}\underset{g\in G}{\inf}d(s,g),\underset{g\in G}{\sup}\underset{s\in S}{\inf}d(s,g)\},$$

where sup represents the supremum, inf the infimum, and *d*(, ) represents the Euclidean distance.

The Dice coefficient is a metric that measures the overlap of the predicted mask and the ground truth mask and varies between 0 (no overlap) to 1 (perfect overlap). The Hausdorff distance, as its name implies is a distance metric indicating the maximal distance between two segmentation masks; thus, smaller values represent better segmentations. We use the 95th percentile of Hausdorff distance to negate the impact of the probable outliers. The 95th percentile Hausdorff distance uses the 95th percentile of the distances between boundary points. The metrics are computed over reassembled 3D volumes. For the WM, GM, and CSF segmentation problem, the Dice and Hausdorff distance are computed per segmentation class and the average value is reported.

To understand how effective is each domain adaptation method in improving the baseline segmentation results, we ran a statistical test. As the assessment of normality of the test results by graphical approaches including histograms and Q-Q plots demonstrated that the distributions are not normal, we chose a non-parametric statistical test. The Wilcoxon signed-rank test with α = 0.05 comparing each DA method for all six domain configurations against the baseline method for each segmentation task. The test was computed using the test samples across all domain configurations to increase the statistical power of the test, since for each domain configuration there are 12 test volumes, and combining all six configurations results in 72 test volumes.

We also report the average percentage of improvement of the DA methods compared to the baseline results. A negative value indicates that the results deteriorated when compared to the baseline models.

### 4.3. Ranking criteria

The methods submitted to the benchmark are ranked separately for each segmentation problem. We chose this approach because we expect to include other brain structures in the benchmark in the future, and also because future submissions to the benchmark may target specific segmentation tasks, thus a ranking per segmentation task is a logical solution.

The rank for each segmentation task is obtained by sorting the results of the DA methods (descending order considering Dice score and ascending order considering Hausdorff distance) for each domain and then calculating how often each method is the best, second best and so on. This approach accounts for the difference in the segmentation quality of the different DA methods across target domains and metrics.

### 4.4. Benchmark methods

#### 4.4.1. Baseline

For the baseline method, we used the U-Net architecture proposed in Ronneberger et al. (2015). The main difference between our implementation and the original U-Net architecture is that we use group normalization (Wu and He, 2018) and dropout after each convolutional layer in our model. Group normalization has been shown to improve model generalization across domains (Perone et al., 2019), and dropout is commonly used to mitigate model overfitting.

The baseline method was trained independently using each domain data for each segmentation problem, resulting in six trained models (i.e., one model for each domain and segmentation problem combination). These six models were tested three times using the source domain and the other two target domains' test data. Each baseline model was trained with early stopping patience set to 5 and using the Adam optimizer with a learning rate of 5 × 10^−4^, which was reduced by half every five epochs. The model dropout rate was set to 0.5. These model hyperparameters were determined empirically.

#### 4.4.2. Fine-tuning final layers

One of the most common supervised domain adaptation methods is fine-tuning a pre-trained model using labeled data in the target domain. The fine-tuning procedure is commonly done on a subset of layers of the model, which are often the final convolutional layers of the model (Ghafoorian et al., 2017). We fine-tune the final two convolutional layers of each baseline model using a fine-tuning set for each target domain. There are three baseline models for each segmentation problem and two target domains, resulting in a total of 12 fine-tuned models. We use 20% of target domain data to fine-tune the last two convolutional layers of the source domain model. This data ratio and the number of fine-tuned layers are chosen empirically based on the experiments conducted in previous work (Shirokikh et al., 2020). The fine-tuning step uses the same training hyperparameters as the baseline models but with an initial learning rate four times smaller (1.25 × 10^−4^).

#### 4.4.3. Fine-tuning first layers

A recent work by Shirokikh et al. (2020) showed that low level feature maps of the input data can be more prone to domain shifts as compared to feature maps in deep layers. Therefore, fine-tuning the first layers of a model could potentially outperform fine-tuning the last layers. For our benchmark, we followed this method-only two initial convolutional layers of the pre-trained model were fine-tuned and the rest of the layers were kept intact. We also used the same hyperparameters proposed by the authors. We did not try fine-tuning the whole model since it has been shown that under scarce data conditions fine-tuning the first layers is superior to fine-tuning the entire model (Shirokikh et al., 2020).

#### 4.4.4. Self-ensembling

We also implemented the self-ensembling method presented in Perone et al. (2019) as an unsupervised method in our benchmark. We chose this method because it has shown promising results for medical image segmentation problems. In principle, this approach employs two different models for self-ensembling: the first model uses a consistency loss between predictions on the same input that has undergone different data augmentation transformations; the second model (temporal ensembling), assumes that as the training advances, averaging the predictions over time on unlabeled samples will help to approximate the actual labels more accurately. During training, this pseudo-label is then used as the target. The consistency loss is minimized along with the cross-entropy for the labeled samples. The network uses the exponential moving average to update the generated targets. Perone et al. (2019) directly combine model weights instead of predictions and call the method Mean Teacher model. We implemented this method, and the code is also in our benchmark code repository. The loss function of this method contains two terms: a term for consistency loss and a second term for segmentation loss. The contribution of the consistency loss in the total loss is controlled by parameter γ. A combination of source domain train set (labeled data) and target domain train set (unlabeled data) serves as the training data for this method. All samples with their respective predictions take part in the training through consistency loss. The labeled data also contribute to segmentation loss when their respective predictions are compared with ground truth masks.

#### 4.4.5. Unlearning: Adversarial unlearning of scanner-variant features

Dinsdale et al. (2021) extended the idea of domain generalization by removing domain-variant features, initially proposed in Ganin et al. (2016) for classification problems, to image segmentation problems. Their unlearning method uses a binary classifier as the domain discriminator attached to a U-Net model as the segmentation network. By attaching a discriminative task like domain classification to the main task (segmentation) and considering an adversarial loss term, the model is encouraged to learn discriminative features for the main task and indiscriminative in regards to the domain classification task. This leads to feature representations that are invariant to where the data was collected while still doing the main segmentation task with minimal performance loss. They achieved promising segmentation results for brain tissue segmentation with T1-weighted brain MRIs acquired by different scanners. We implemented the unlearning method for both of our segmentation tasks by training a three-part network consisting of a domain classifier with parameters θ_*d*_, a feature extractor with parameters θ_*repr*_, and a segmentation network with parameters θ_*p*_ which does our main segmentation task using the features extracted by the feature extractor network. We used our baseline U-Net model for the feature extractor and segmentation network. The total loss of the method is composed of three terms: a term used in the first stage to pre-train the segmentation task, a term to tune the domain classifier's parameters, and a confusion loss term to tune the θ_*repr*_ in such a way to remove the domain variant features from the feature extractor results so it can confuse the domain classifier in distinguishing from which domain the data come from. We use the Dice score for the segmentation loss, categorical cross-entropy for the domain classifier loss, and sum of log losses for the confusion loss. The contribution of the domain classifier loss and the confusion loss is controlled by weighting the corresponding loss terms with parameters α and β which have negative values to make adversarial training possible. This method can be implemented both as an unsupervised method using unlabeled target domain data for training the domain predictor and using only the source labeled data for learning the segmentation masks, or as a semi-supervised method by using some labeled target domain data for training the segmentation network too. We used the semi-supervised approach proposed by Dinsdale et al. (2021) in this benchmark.

### 4.5. Implementation details

The benchmark code is implemented in Python 3.6.8, and PyTorch 1.9.1 is used as the deep learning framework. We trained our models on an NVIDIA Tesla V100 GPU with 16 GB memory. The training times vary based on the method, segmentation task and domains. For the baseline method, each epoch takes about 243 s to run. Fine-tuning first layers takes 216 s. For fine-tuning last-layers, it is 227 s, 850 s for the self-ensembling method, and 450 s for the unlearning method for skull-stripping segmentation. The same methods for WM, GM, and CSF fluid segmentation take 304, 203, 180, 923, and 688 s to run per epoch, respectively. To avoid overfitting, we set an early stopping controller for 25 epochs. All methods are trained with batch size of 16. The hyperparameters of the fine-tuning methods and the self-ensembling method are set to the suggested values proposed in their original publications. We investigated tuning them, but we did not see improvement in the DA results. We initially tried employing the hyperparameters proposed by the authors for the unlearning models, but they either failed to converge or achieved poor results. For this reason, we conducted a grid search on the hyperparameters space (α and β) to optimize the DA capabilities of the models. The grid search was run for all domains for both segmentation tasks individually on the interval of [5, 60] with step size of 5. The best hyperparameters we found were α = 30 and β = 30 for all configurations and segmentation tasks.

### 4.6. New submissions

Our benchmark, including the implementation of the above methods and the code to extract the metrics and generate the ranking of the different methods, is publicly available on the benchmark repository. Our benchmark accepts new submissions from independent research groups. A list of test volumes is provided within the benchmark. Researchers interested in submitting methods to our benchmark can submit their predicted segmentation masks for this test data, and our team will process the results and make an update to the benchmark leaderboard (https://www.ccdataset.com/brain-mri-segmentation-playground). Our code repository provides instructions that allow researchers to reproduce the exact same experimental setup that we use here, thus making the comparison of new future submissions objective and fair.

## 5. Results

**Figure 3:**
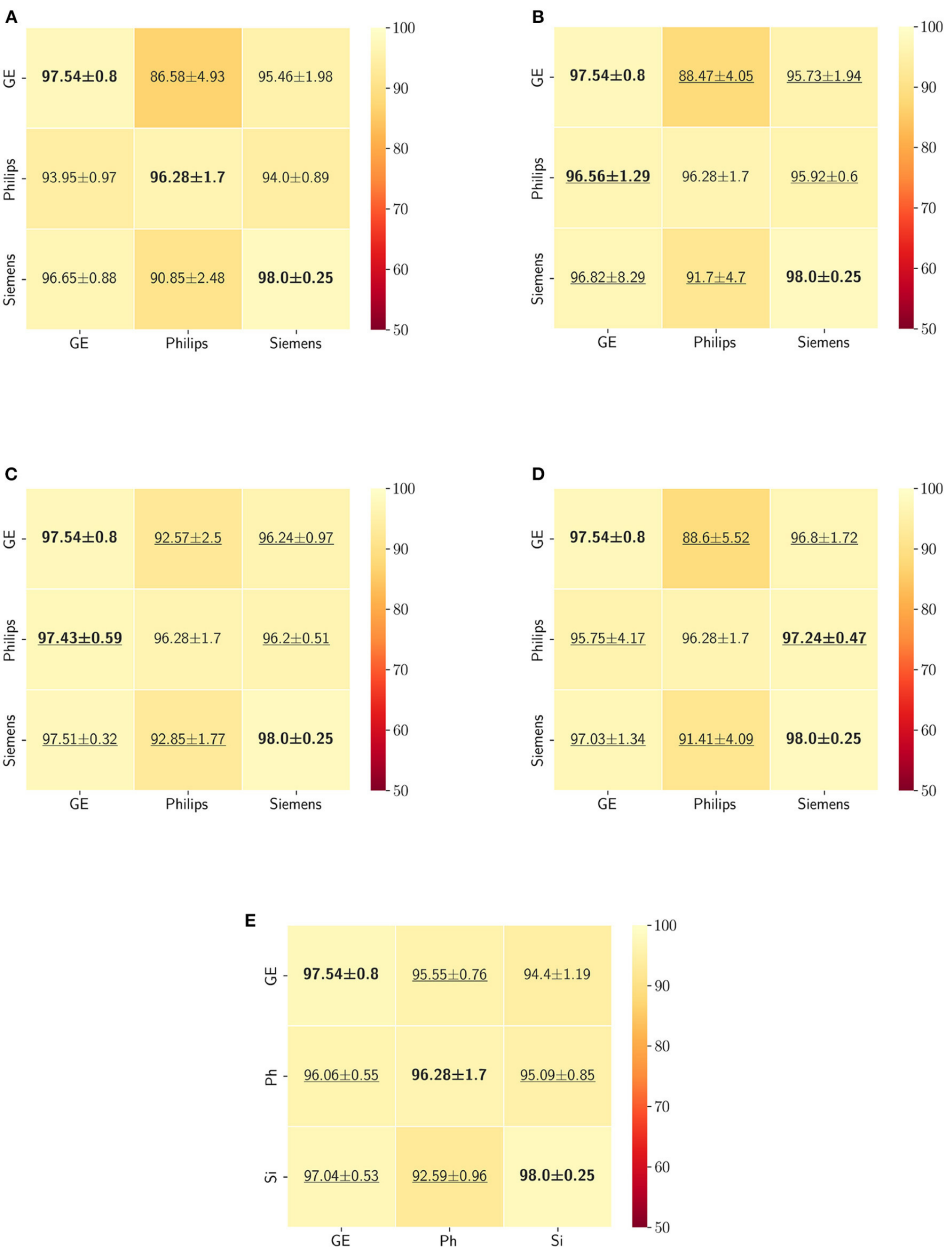
Dice coefficient results (mean ± standard deviation) for the skull-stripping segmentation displayed as heatmaps. The main diagonal values in the heatmaps **(B–E)** are from the baseline models since the source and the target domains are the same. The cases where the DA model improved the results compared to the baseline are underlined and the best score each model achieved is shown in bold. **(A)** Baseline, **(B)** fine-tuning last layers, **(C)** fine-tuning first layers, **(D)** self-ensembling, and **(E)** unlearning.

**Figure 4:**
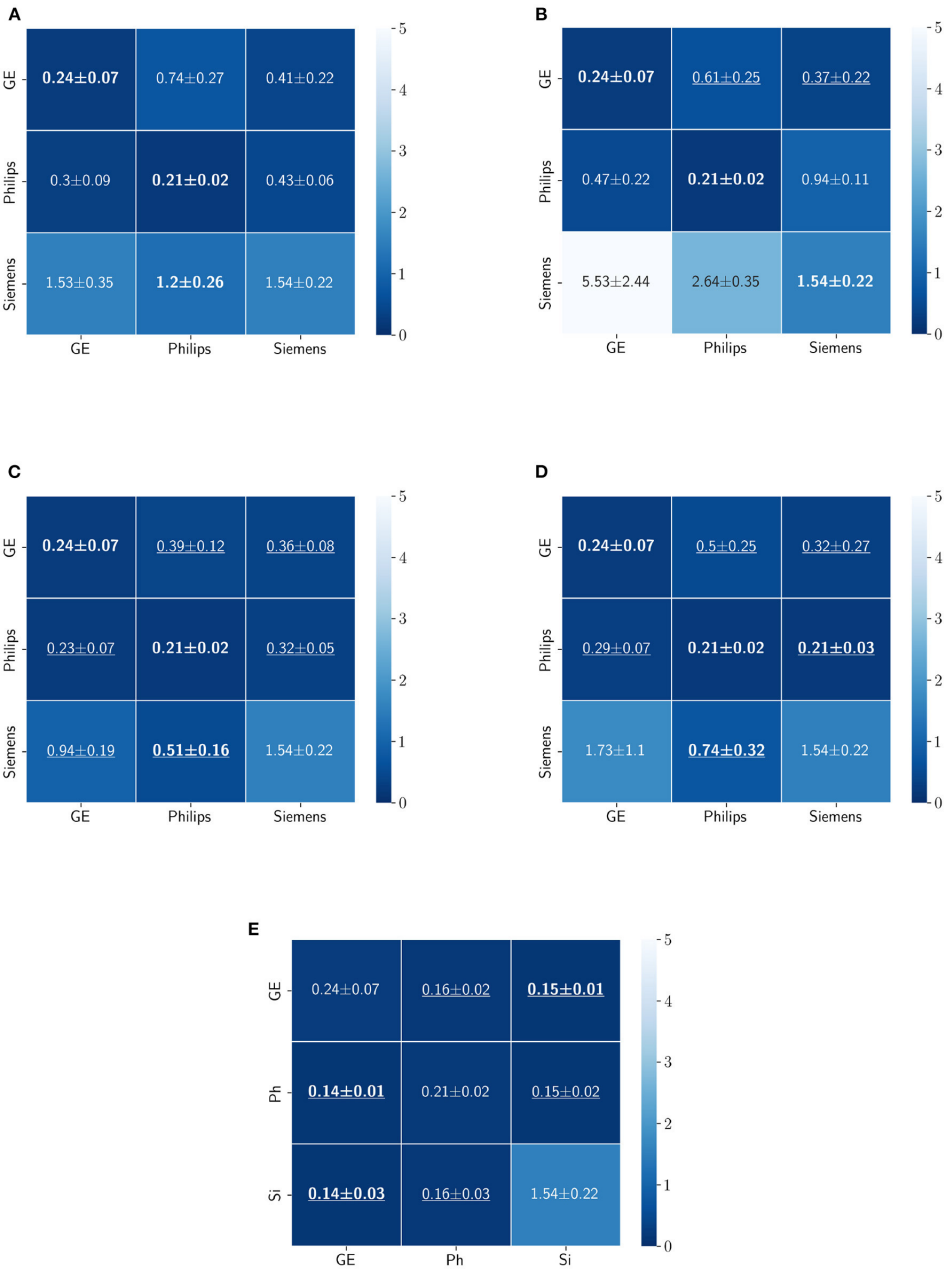
Haussdorff distance results (mean ± standard deviation) for the skull-stripping segmentation displayed as heatmaps. The main diagonal values in the heatmaps **(B–E)** are from the baseline models since the source and the target domains are the same. The cases where the DA model improved the results compared to the baseline are underlined and the best score each model achieved is shown in bold. **(A)** Baseline, **(B)** fine-tuning last layers, **(C)** fine-tuning first layers, **(D)** self-ensembling, **(E)** unlearning.

The Dice coefficient and Hausdorff distance results for the skull-stripping segmentation problem are summarized in Figures 3, 4, respectively. The rows in these figures represent the domain which the model is trained on, and the columns represent the test domains. By comparing the results of the baseline Dice score and DA methods, we see that the DA methods compensate for the domain shift in most scenarios. For the Dice coefficient metric, the unlearning Dice metric slightly decreased when compared to the baseline model when the source domain data are from GE and the target domain data are from Siemens. For the Hausdorff distance metric, the fine-tuning last layers models only improved the results in two out of the six domain configurations, unlearning improved in five domain configurations, while fine-tuning first layers and self-ensembling improved the results in all domain configurations.

The ranking of the DA methods and the percentage of improvement is depicted in Table 3. The ranking for the Dice coefficient metric shows that fine-tuning the first layers of U-Net is more often generating better results and is ranked first among the DA methods in 50% of cases. Self-ensembling is the best DA method in 33.33% of cases. The unlearning method was shown to be the best in 16.67% of the cases, while fine-tuning the last layers was not ranked first in any of the domain configurations.

**Table 3:** Summary of the results.

		**Skull-stripping**				**WM, GM, CSF**			

		**FT last layers (%)**	**FT first layers (%)**	**Self-ensembling (%)**	**Unlearning (%)**	**FT last layers (%)**	**FT first layers (%)**	**Self-ensembling (%)**	**Unlearning (%)**
Dice score	Improvement %	1.40	**2.81**	1.68	2.5	30.19	**31.34**	30.69	31.05
	Ranked 1st	0	50	33.33	16.67	33.33	50	0	16.67
	Ranked 2nd	16.67	50	0	33.33	16.67	33.33	0	50
	Ranked 3rd	33.33	0	50	16.67	16.67	16.67	66.67	0
	Ranked 4th	50	0	16.67	33.33	33.33	0	33.33	33.33
Haussdorf distance	Improvement %	−88.23	34.08	22.36	**72.96**	1.24	39.32	42.46	59.95
	Ranked 1st	0	16.67	33.33	50	0	0	16.67	83.33
	Ranked 2nd	0	50	0	50	33.33	16.67	50	0
	Ranked 3rd	0	33.33	66.67	0	33.33	50	0	16.67
	Ranked 4th	100	0	0	0	83.33	0	16.67	0

The improvement percentage is the average metric improvement obtained by the DA method in relation to the baseline method and negative values represents that the DA method had worse results comparing to the baseline method. The methods with highest improvement percentage are highlighted in bold. Rank first, second, and third represent the percentage of cases each DA method was ranked first, second, or third, respectively.

**Figure 5:**
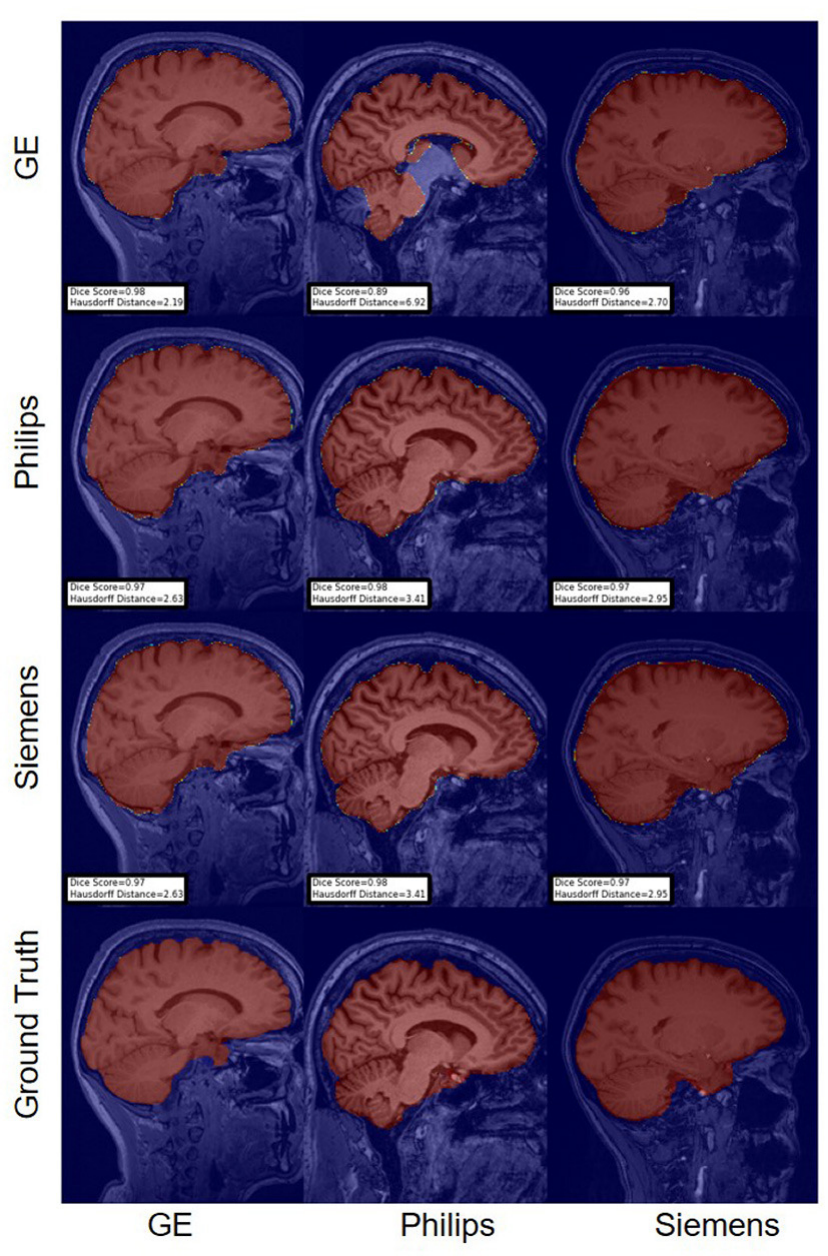
Representative sample skull-stripping segmentation generated by the baseline models. Rows indicate the source domain which the model is trained on and columns indicate the target domain on which the model is tested. The first row from the bottom indicates the ground truth mask.

**Figure 6:**
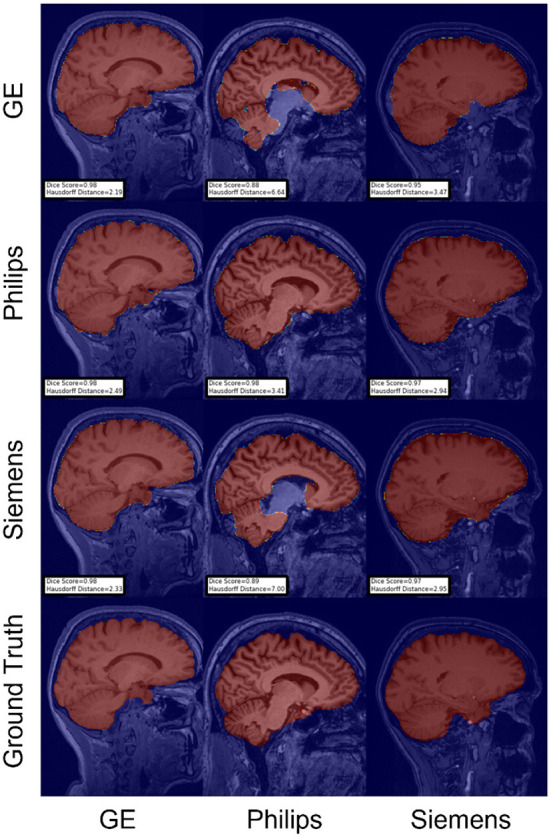
Representative sample skull-stripping segmentation generated by fine-tuning the last layers of the U-Net. Rows indicate the source domain which the model is trained on and columns indicate the target domain on which the model is tested. The first row from the bottom indicates the ground truth mask.

**Figure 7:**
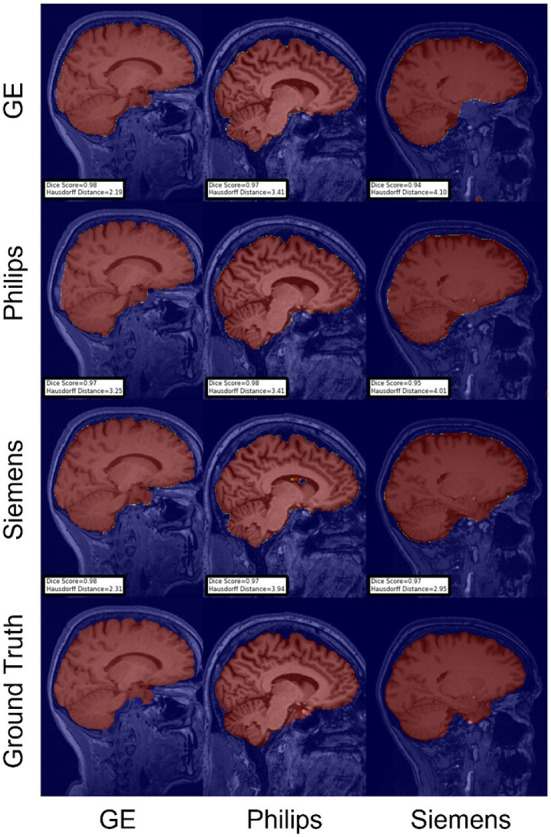
Representative sample skull-stripping segmentation generated by fine-tuning the first layers of the U-Net. Rows indicate the source domain which the model is trained on and columns indicate the target domain on which the model is tested. The first row from the bottom indicates the ground truth mask.

**Figure 8:**
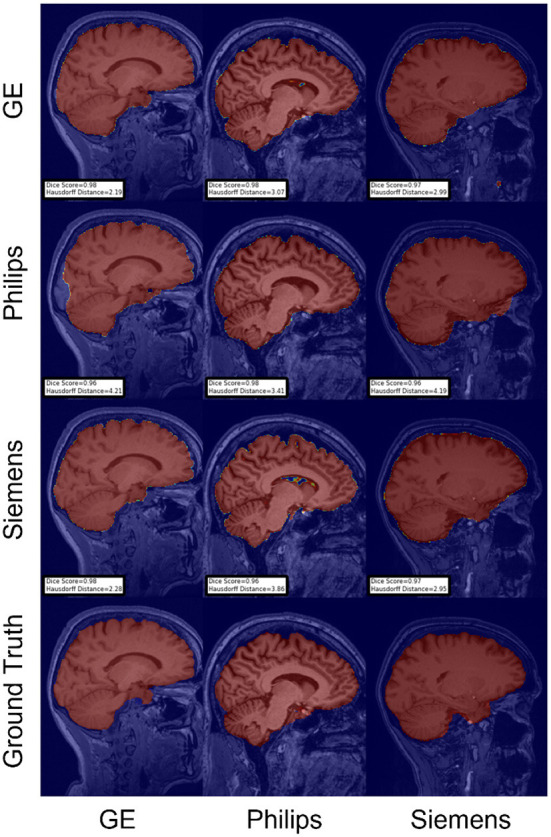
Representative sample skull-stripping segmentation generated by the self-ensembling. Rows indicate the source domain which the model is trained on and columns indicate the target domain on which the model is tested. The first row from the bottom indicates the ground truth mask.

**Figure 9:**
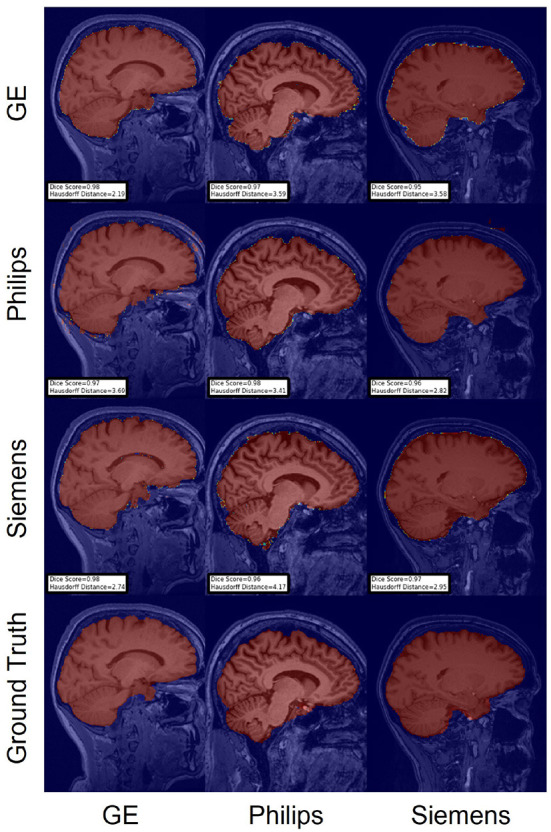
Representative sample skull-stripping segmentation generated by unlearning. Rows indicate the source domain which the model is trained on and columns indicate the target domain on which the model is tested. The first row from the bottom indicates the ground truth mask.

For the Hausdorff distance results, we can see that unlearing has the greatest percentage of improvement (72.96%) followed by fine-tuning the first layers (34.08%). The self-ensembling method showed an improvement of 22.36%. Fine-tuning the last layers had a negative percentage of improvement (−88.23%). The unlearning method ranked first in 50% of cases among all DA methods in terms of improving Hausdorff distance, followed by the self-ensembling methods, which ranked first in 33.33% of the cases, and then the fine-tuning the first layers of the U-Net, which was ranked first in 16.67% of the cases. Fine-tuning the last layers of the U-Net has always ranked as the last method in improving the Hausdorff distance. Sample segmentations for the baseline, fine-tuning final layers, fine-tuning first layers, self-ensembling, and unlearning methods are summarized in Figures 5–9, respectively. In these figures, we can see clear segmentation failures for the baseline model trained on GE data and tested on Philips data that all DA methods, except for fine-tuning last layers, were able to improve.

**Figure 10:**
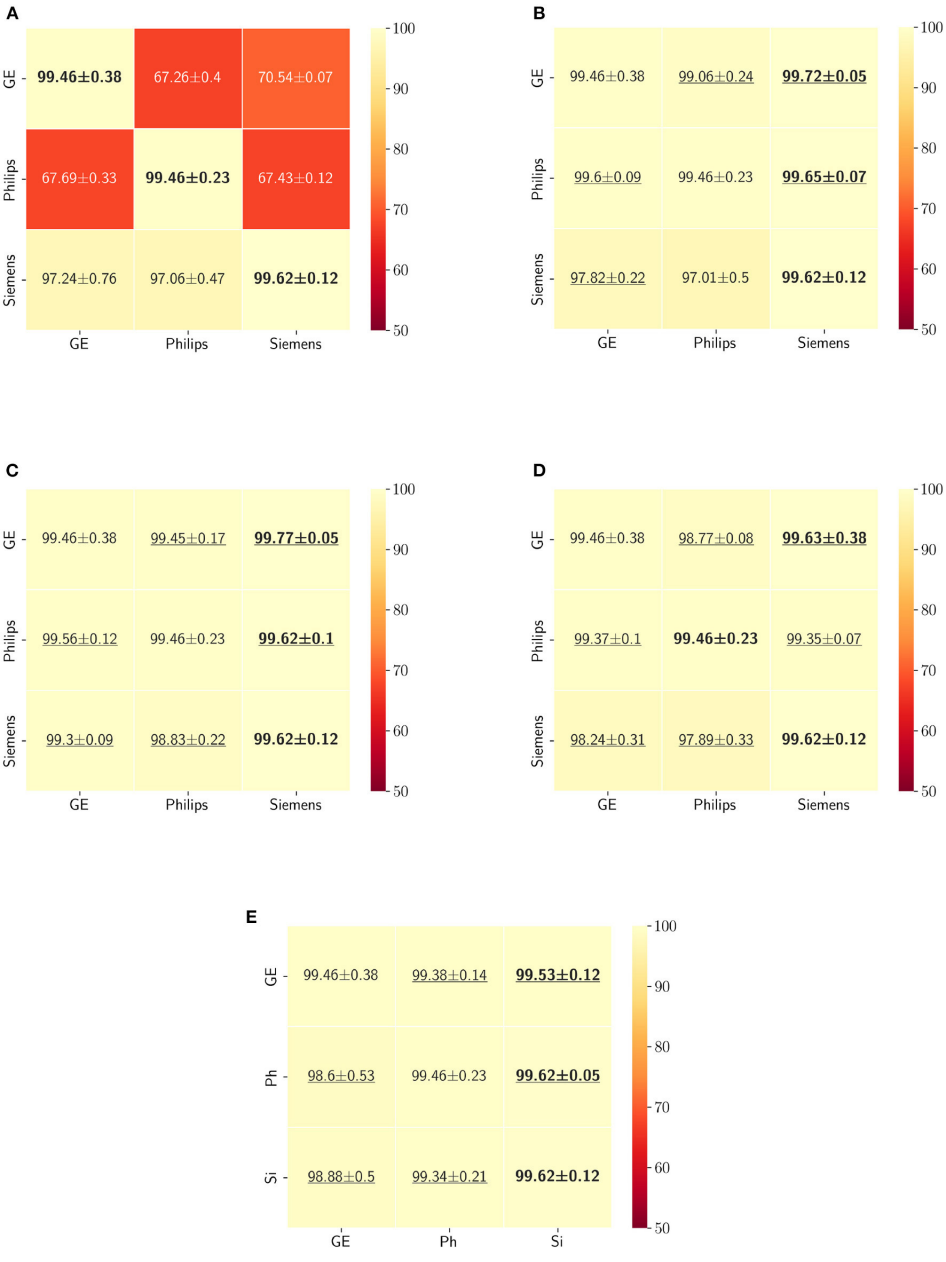
Dice coefficient results (mean ± standard deviation) for the WM, GM, and CSF segmentation displayed as heatmaps. The main diagonal values in the heatmaps **(B–E)** are from the baseline models since the source and the target domains are the same. The cases where the DA model improved the results compared to the baseline are underlined and the best score each model achieved is shown in bold. **(A)** Baseline, **(B)** fine-tuning last layers, **(C)** fine-tuning first layers, **(D)** self-ensembling, and **(E)** unlearning.

**Figure 11:**
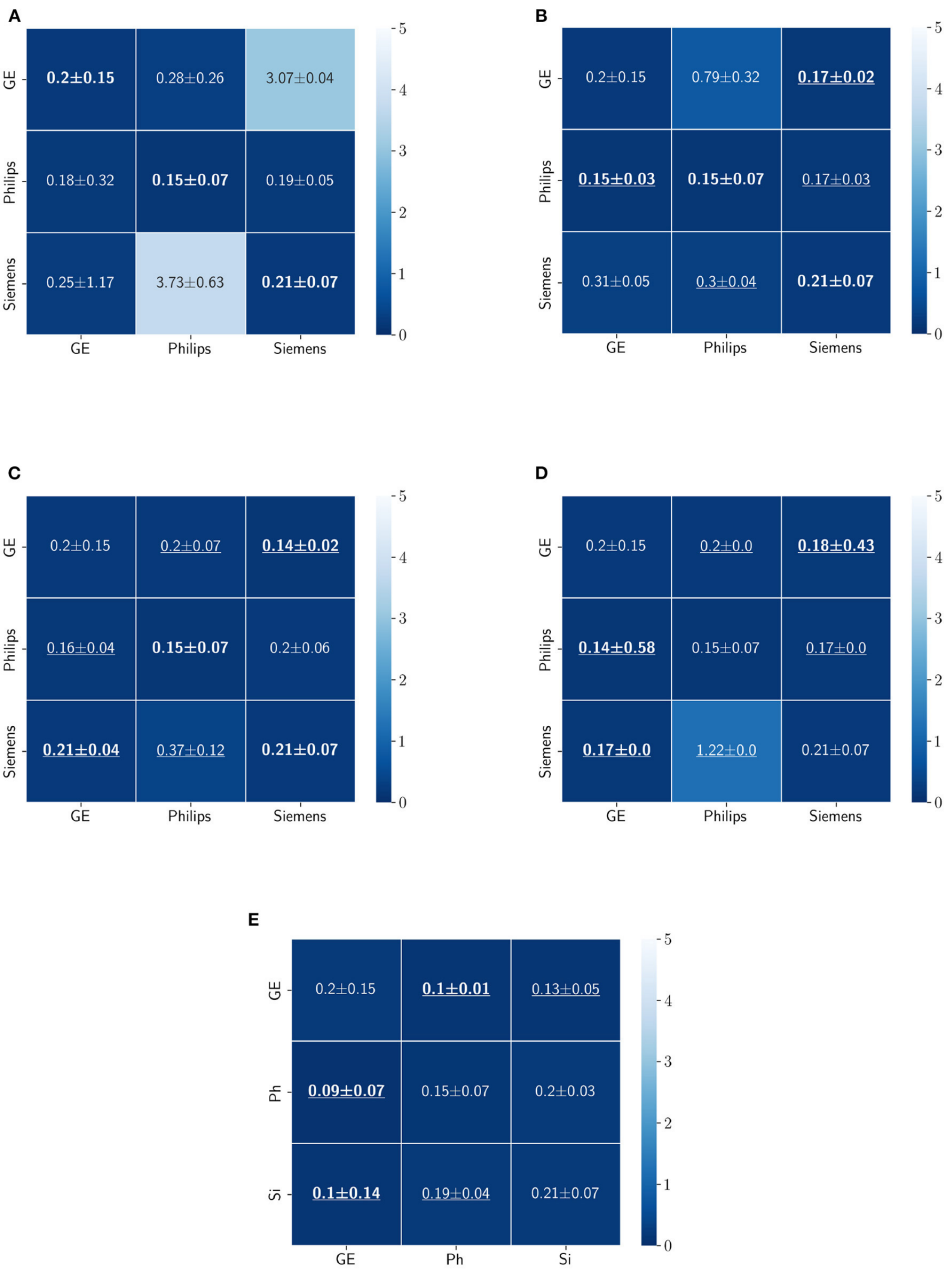
Haussdorff distance results (mean ± standard deviation) for WM, GM, and CSF segmentation displayed as heatmaps. The main diagonal values in the heatmaps **(B–E)** are from the baseline models since the source and the target domains are the same. The cases where the DA model improved the results compared to the baseline are underlined and the best score each model achieved is shown in bold. **(A)** Baseline, **(B)** fine-tuning last layers, **(C)** fine-tuning first layers, **(D)** self-ensembling, and **(E)** unlearning.

**Figure 12:**
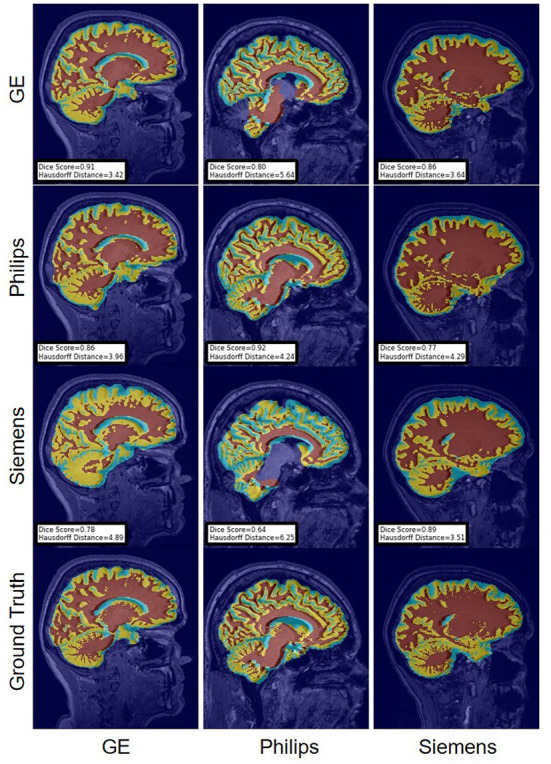
Representative sample WM, GM, CSF segmentation generated by the baseline models. Rows indicate the source domain which the model is trained on and columns indicate the target domain on which the model is tested. The first row from the bottom indicates the ground truth mask.

**Figure 13:**
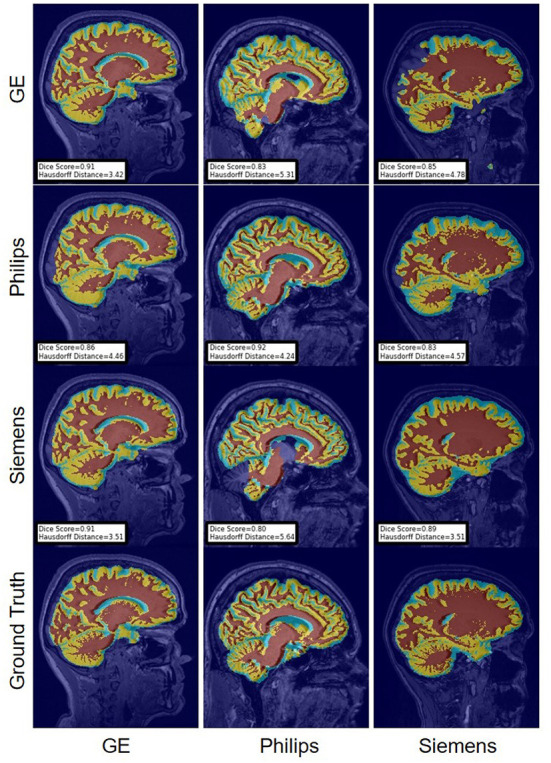
Representative sample WM, GM, CSF segmentation generated by fine-tuning the last layers of the U-Net models. Rows indicate the source domain which the model is trained on and columns indicate the target domain on which the model is tested. The first row from the bottom indicates the ground truth mask.

**Figure 14:**
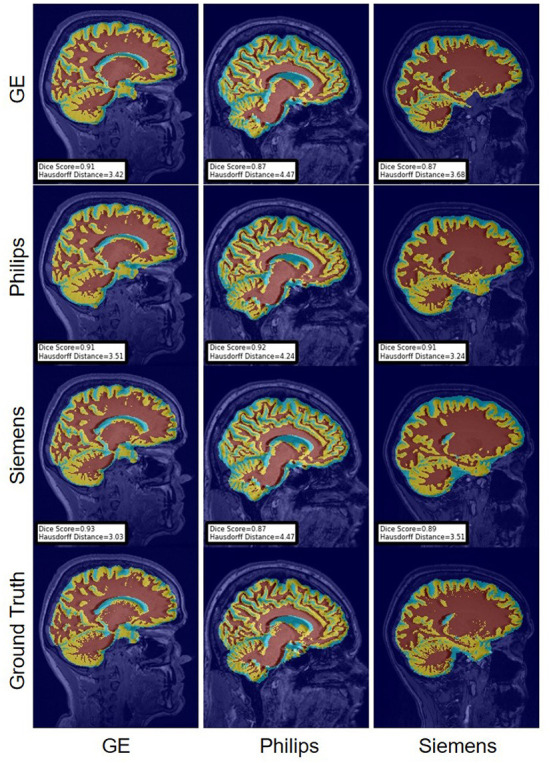
Representative sample WM, GM, CSF segmentation generated by fine-tuning the first layers of the U-Net models. For the scenarios where the train and the test domains are the same, we display the baseline models' results. Rows indicate the source domain which the model is trained on and columns indicate the target domain on which the model is tested. The first row from the bottom indicates the ground truth mask.

**Figure 15:**
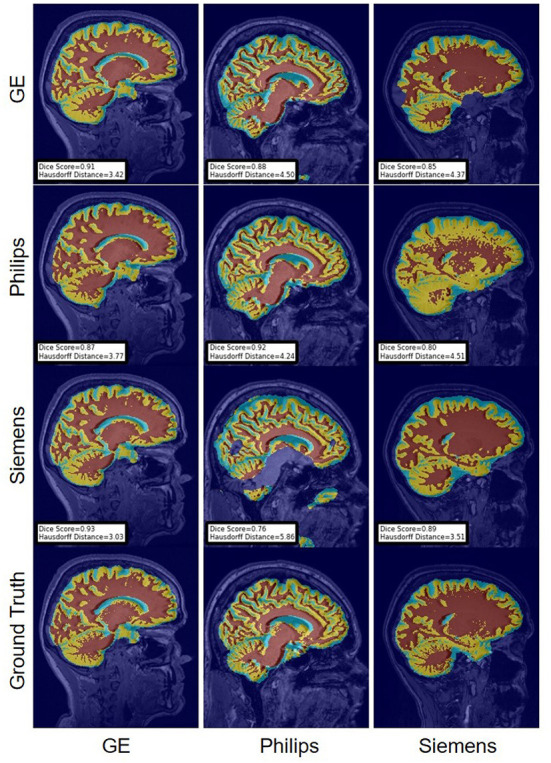
Representative sample WM, GM, CSF segmentation generated by the self-ensembling models. For the scenarios where the train and the test domains are the same, we display the baseline models' results. Rows indicate the source domain which the model is trained on and columns indicate the target domain on which the model is tested. The first row from the bottom indicates the ground truth mask.

**Figure 16:**
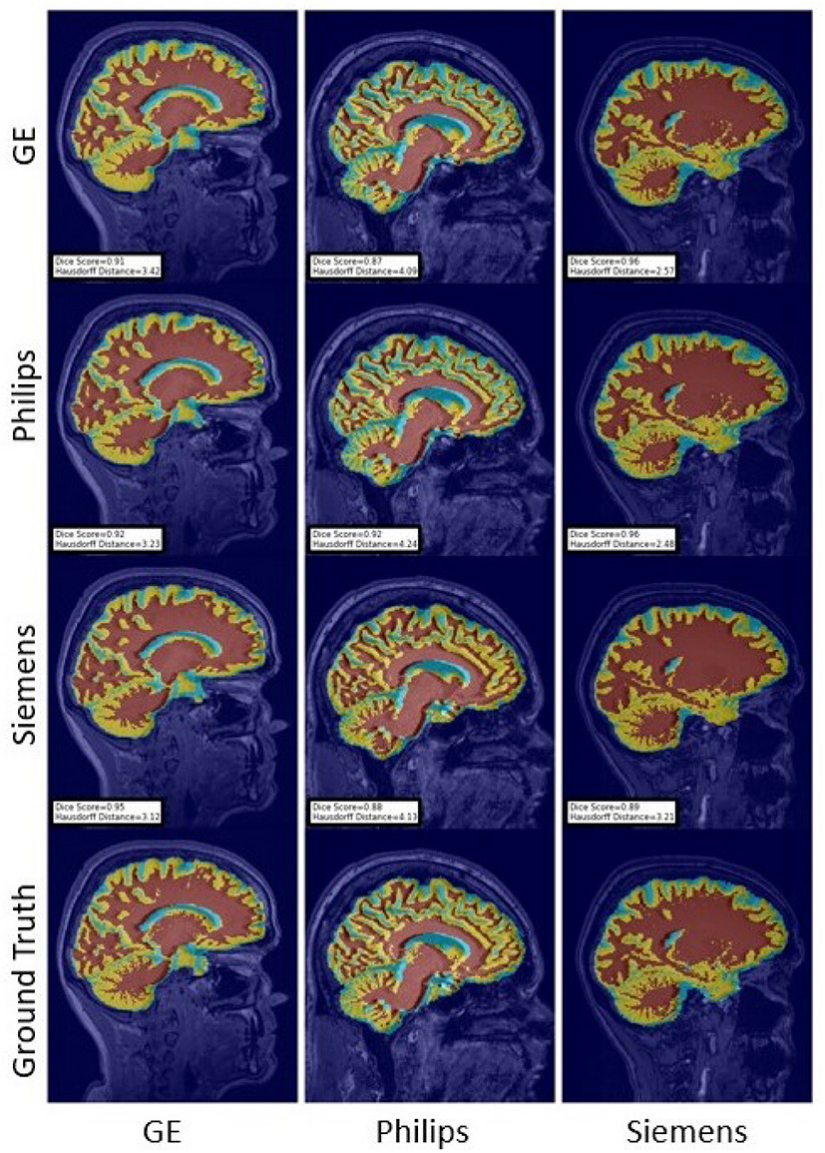
Representative sample WM, GM, CSF segmentation generated by the unlearning models. For the scenarios where the train and the test domains are the same, we display the baseline models' results. Rows indicate the source domain which the model is trained on and columns indicate the target domain on which the model is tested. The first row from the bottom indicates the ground truth mask.

The Dice coefficient and Hausdorff distance results for the WM, GM, CSF segmentation problem are summarized in Figures 10, 11, respectively. The rows in these figures represent the domain which the model is trained on, and the columns represent the test domains. Fine-tuning the first layers of U-Net showed high performance in improving the segmentation results of the WM, GM, CSF segmentation in terms of Dice score by being the best method in 50% of domain configuration cases. The unlearning method ranked first at 83.33% of scenarios in terms of improving the Hausdorff distance. Sample segmentations for the baseline, fine-tuning final layers, fine-tuning first layers, self-ensembling, and unlearning methods are summarized in Figures 12–16, respectively. These figures show segmentation failures for some of the DA methods for some specific target domain sets.

**Figure 17:**
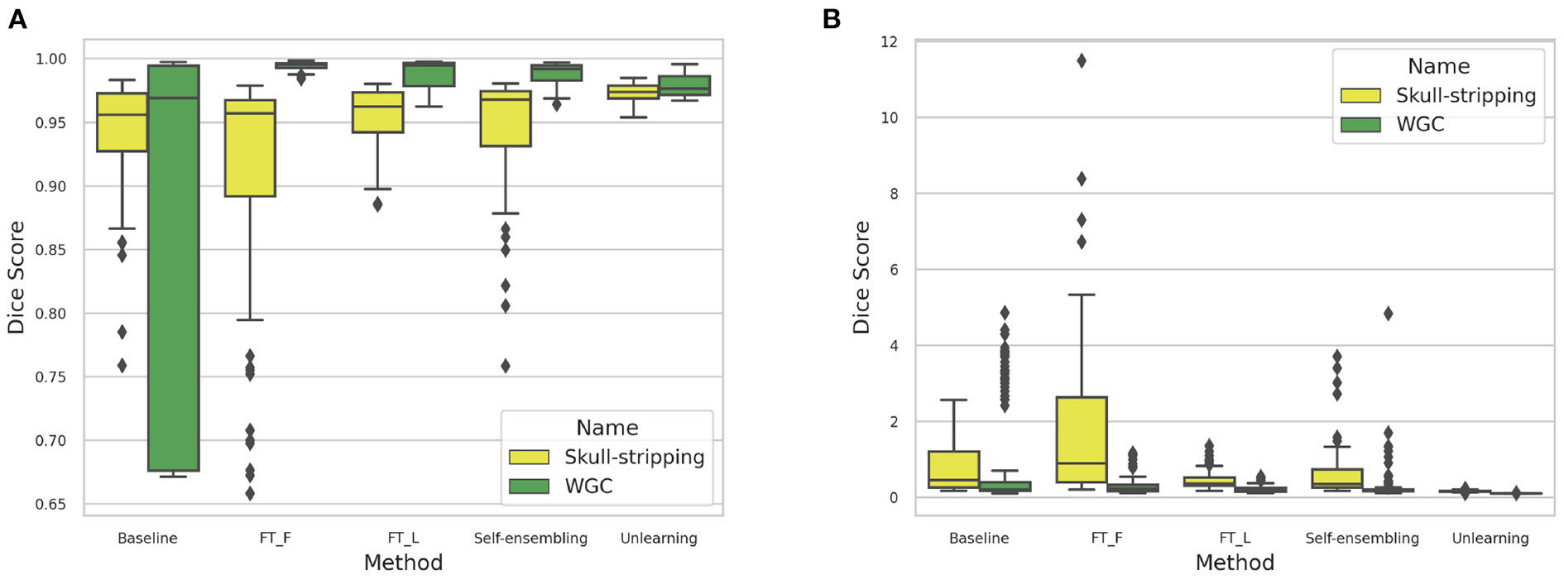
**(A)** Dice coefficient and **(B)** Haussdorff distance box-plot distributions computed for the different methods across the 6 target domains for the skull-stripping and WM, GM, and CSF segmentation problems.

The DA results are summarized in Table 3. Box-plots highlighting the Dice coefficient and Hausdorff distance results for the skull-stripping and WM, GM, CSF segmentation problems for the different domains are depicted in Figure 17.

Cause the distribution of the test results are not normal, we chose a non-parametric statistical test to assess the effectiveness of DA methods in resolving the domain shift. The Wilcoxon signed-rank statistical significance test was computed for each method per segmentation task and per segmentation metric, leading to a total of 16 test results. In all cases, the differences were deemed to be significant (*p* < 0.05). In 14 cases, *p* < 0.001, and in the other two *p* < 0.005.

## 6. Discussion

### 6.1. Benchmark

In the present work we addressed the problem of absence of objective metrics that can compare performance of different domain adaptation (DA) techniques. We built a DA benchmark for MRI brain segmentation using the CC-359 dataset, which consists of three data domains. The benchmark currently consists of a baseline model with no DA strategy along with four DA methods. Three supervised DA methods based on fine-tuning either the first or last layers of models pre-trained using source domain data, and another method based on unlearning domain specific features. One unsupervised DA method that uses a self-ensembling approach. Our results showed that the performance of the baseline segmentation model degrades across domains, and employing DA methods can be used to improve results. The fine-tuning of the first layers of the baseline was ranked first in most of the target domain results considering the Dice score as the evaluation metric, while based on the Haussdorf distance metric the unlearning method ranks first in most cases.

Fine-tuning pre-trained models is relatively easy to implement and fast to run. Fine-tuning also has the advantage that access to source domain data os not needed. The unlearning model aims to create scanner-invariant features using an iterative training scheme based on domain adaptation techniques, whilst simultaneously completing the desired segmentation task. The limitation of these methods is the necessity of having labeled data in the target domain, which may not always be available. The self-ensembling DA method does not require labeled data in the target domain, hence is more desirable for medical applications. However, it is a more complex model and slower to train compared to fine-tuning and unlearning.

The purpose of this benchmark was to provide labeled data, open source code for a set of baseline models, and a benchmark for comparing different brain MR image segmentation techniques. However, we experienced various difficulties along the way. Most methods we have used in the paper did have their source code but we had to adapt them to our dataset. The hyperparameter tuning was also difficult as our dataset has three domains (GE, Philips, Siemensi) and two tasks [skull-stripping (SS), WM, GM, CSF segmentation], which results in training and hyperparameter tuning of 12 different models.

The source code for most of the domain adaptation methods other than the ones mentioned in our work was unavailable. The majority of the works' implementation details are also insufficient, making the process of writing the code ourselves significantly more time-consuming. Most techniques also needed hyperparameter calibration, as details are missing from the respective papers. We tried many alternative ways in addition to those described in this study, but the results were poor for the reasons mentioned. We believe that our work will greatly enhance the transparency and accessibility of scientific research in the field of neuroscience. For all of the approaches discussed in the study, we have kept publicly available data as well as an up-to-date code repository accessible. We also have a webpage with all of the information and hyperparameter settings for smoother navigation of our work.

### 6.2. Skull-stripping

As Table 3 reports, fine-tuning the first layer of the U-Net method is often ranked first among the other DA methods (in 50% of scenarios), followed by the self-ensembling method (in 33.33% of scenarios) and unlearning method (in 16.67% of scenarios) while fine-tuning the last layers of U-Net never shows up as the best method. The greatest percentage of improvements in terms of both average Dice score and average Haussdorf distance belongs to the unlearning method.

The lower performance of fine-tuning the last layers of U-Net could be interpreted as evidence to support the assumption that the first layers of a model contain more domain-specific information (Shirokikh et al., 2020) and the last layers are dealing with higher-level features, almost independent of the data domain. Therefore, fine-tuning the first layers of the U-Net eliminates the effect of having features dependent on the source domain data distribution and can be used to tune pre-trained models to the target domain distribution.

### 6.3. WM, GM, CSF segmentation

WM, GM, CSF Segmentation is a multi-class segmentation problem and more complex than the skull-stripping problem. Based on the results, fine-tuning the first layers of U-Net is the best DA method to resolve the domain shift since it is ranked first (50% of cases) among all three DA methods, based on the Dice score. Fine-tuning the last layers of U-Net also improves the baseline performance, but the improvements are not as effective as fine-tuning the first layers. Self-ensembling also is the first ranked method in 33.33% of cases. Considering the Haussdorf distance metric, the unlearning method has the most improvement percentage and also is mostly ranked first (83.33% of cases) among all other methods.

## 7. Conclusions

In this work, we proposed a benchmark for evaluating different DA methods for brain MRI segmentation. Our benchmark and the CC-359 dataset used to develop our method are publicly available. Therefore, our benchmark can be used by other researchers for the assessment of novel DA methods. The benchmark consists of baseline, supervised, and unsupervised DA methods to cover different possible approaches. We showed that both supervised and unsupervised DA methods are effective in addressing the issue of domain shifts. Fine-tuning the first layers of the U-Net has outperformed the fine-tuning the last layers, unlearning, and also the self-ensembling method in both skull-stripping and WM, GM, and CSF segmentation regarding the improvements of the dice score. Nevertheless, the unlearning method has achieved the best improvement in terms of the Haussdorf distance metric. This finding associated with difficulties in hyperparameter tuning, long training times of models, such as unlearning, are an obstacle to practical adoption of these methods and present an interesting research venue that has been little explored.

Different segmentation tasks and DA methods can be added to the benchmark to create a comprehensive platform for evaluating different brain MRI segmentation methods' performance, and determining, which methods are more robust in terms of generalizability across different data domains. Future research works should aim for adding more DA methods to the benchmark and developing an ensemble method that can choose the best DA method for each data domain.

## Data availability statement

Publicly available datasets were analyzed in this study. This data can be found at: www.ccdataset.com.

## Author contributions

PS implemented the algorithms and wrote the manuscript. NN was responsible for reviewing the WM, GM, and CSF segmentations. MG and MH implemented the unlearning algorithm, investigated image translation methods, and helped with the figures and response letter. RS and HH provided the funding to develop the work. All authors actively participated in the design of the experiments and in reviewing the submitted manuscript. All authors contributed to the article and approved the submitted version.

## Conflict of interest

The authors declare that the research was conducted in the absence of any commercial or financial relationships that could be construed as a potential conflict of interest.

## Publisher's note

All claims expressed in this article are solely those of the authors and do not necessarily represent those of their affiliated organizations, or those of the publisher, the editors and the reviewers. Any product that may be evaluated in this article, or claim that may be made by its manufacturer, is not guaranteed or endorsed by the publisher.

## Footnotes

^1^ https://github.com/ParisaSaat/Brain-MR-Segmentation-Playground

^2^ https://www.ccdataset.com/brain-mri-segmentation-playground
