# Supplementary material for: A domain adaptation benchmark for T1-weighted brain magnetic resonance image segmentation
Source: Front Neuroinform. 2022 Sep 23;16:919779. doi: 10.3389/fninf.2022.919779 (PMC9538795; doi:10.3389/fninf.2022.919779)
Supplement: Supplementary file 1 [file Table_1.DOCX]

**Supplementary Material**

**
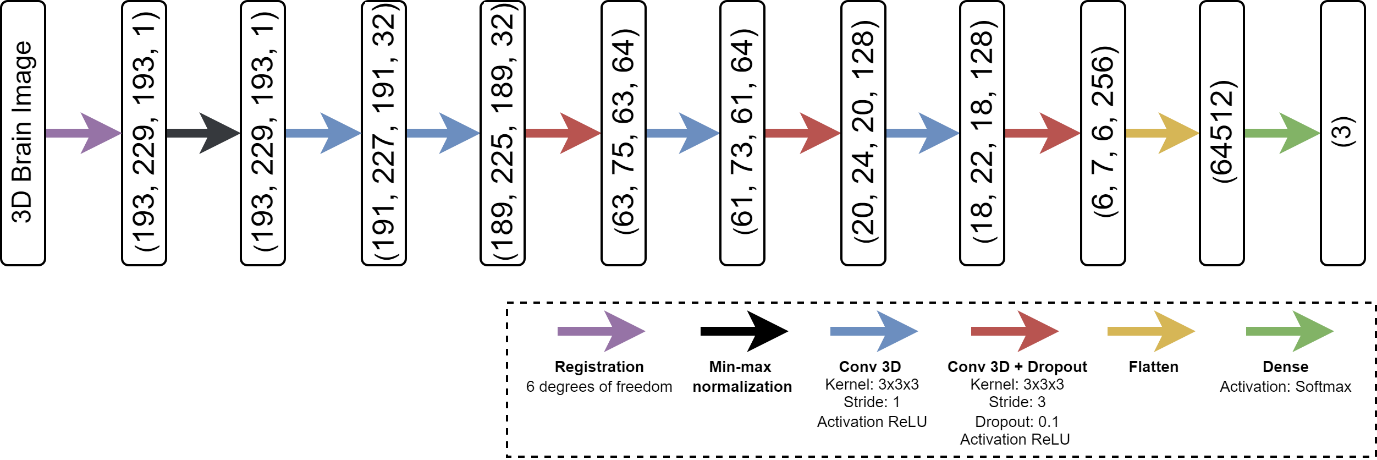
**

To justify our choice of three different domains (Philips, Siemens and General Electric) in our benchmark, we developed a simple classification model to detect how easily this model can distinguish between these three domains. The overall approach is described in the figure above. Initially, each 3D image volume is registered to the ICBM 2009a Nonlinear Symmetric atlas [1] using a 6-degree-of-freedom linear registration implemented in the FLIRT command of the FSL software [2]. Then, the 3D Convolutional Neural Network shown in the figure above is trained. The accuracy results of a 5-fold cross-validation procedure resulted in 99.44% ± 0.79 (mean ± std) accuracy, thus supporting our decision of using MR images from each scanner vendor as a separate domain.

[1] V.S. Fonov, A.C. Evans, K. Botteron, C.R. Almli, R.C. McKinstry, D.L. Collins and B.D.C.G., Unbiased average age-appropriate atlases for pediatric studies, NeuroImage, 54, 2011.

[2] M.W. Woolrich, S. Jbabdi, B. Patenaude, M. Chappell, S. Makni, T. Behrens, C. Beckmann, M. Jenkinson, S.M. Smith. Bayesian analysis of neuroimaging data in FSL. NeuroImage, 45:S173-86, 2009
